# Clinical and operative risk factors for complications after Apert hand syndactyly reconstruction

**DOI:** 10.1177/17531934231213516

**Published:** 2023-11-21

**Authors:** Holly Cordray, Emily M. Graham, Anchith Kota, Apurva S. Shah, Benjamin Chang, Shaun D. Mendenhall

**Affiliations:** 1 Perelman School of Medicine at the University of Pennsylvania, Philadelphia, PA, USA; 2 Division of Plastic, Reconstructive, and Oral Surgery, Children’s Hospital of Philadelphia, Philadelphia, PA, USA; 3 Division of Orthopaedic Surgery, Children’s Hospital of Philadelphia, Philadelphia, PA, USA

**Keywords:** Acrocephalosyndactylia, Apert syndrome, Apert hand, syndactyly, hand surgery

## Abstract

This study evaluated how Apert hand syndactyly presentations and reconstructive techniques influence reconstruction outcomes. All cases at a major paediatric hospital between 2007 and 2022 were analysed, including 98 web space reconstructions in 17 patients. Overall, 62% of hands developed complications and 15% required revision surgery. Upton hand type was significantly associated with postoperative complication incidence, specifically including range-of-motion deficits, flexion contracture, web creep and revision surgery. More severe syndactylies may benefit from additional measures to reduce complications. Rectangular commissural flaps showed 1.9 times greater complication risk than interdigitating triangular flaps, including 11.2 times greater risk of web creep. Zigzag volar finger flaps showed 1.8 times greater complication risk than straight-line incisions, including 3.8 times greater risk of web creep. Our study showed that interdigitating triangular commissural flaps and straight-line volar finger incisions are preferable to rectangular commissural and zigzag finger flaps in most cases of Apert hand syndactyly to minimize complications.

**Level of evidence:**

III

## Introduction

The hands in Apert syndrome display complex, symmetrical syndactyly, which accompanies craniosynostosis, midface hypoplasia and lower-extremity syndactyly. The syndrome arises from a substitution mutation in the fibroblast growth factor receptor 2 (FGFR2) gene and affects approximately one in 65,000 live births (Cohen et al., 1992; Lajeunie et al., 1999). Apert hands typically demonstrate bony or cartilaginous fusion of the index, long and ring fingers, simple syndactyly of the fourth web space and possible syndactyly of the thumb with radial clinodactyly (Guero et al., 2004; Upton, 1991). Concomittant symphalangism at the proximal interphalangeal joints of the fingers and of the interphalangeal joint of the thumb may also occur, often requiring functional osteotomies during childhood to restore hand function (Fearon, 2003; Guero et al., 2004).

The Upton classification system (Upton, 1991) defines three types of Apert hands according to increasing severity. Type I involves fusion of the three central digits and a shallow first web space with a largely free thumb in a ‘spade’ conformation, Type II involves a concave palm due to fusion of the three central digits and thumb in a ‘mitten’ conformation and Type III involves tight fusion of all five digits in a ‘rosebud’ conformation (Upton, 1991).

Phenotypic diversity in Apert syndrome has caused variability and controversy over treatment approaches (Chang et al., 2002; Fearon, 2003; Fearon and Podner, 2013; Guero, 2005; Lajeunie et al., 1999; Pettitt et al., 2017; Raposo-Amaral et al., 2018; Van Heest et al., 1997). The multisystemic nature of the syndrome demands extensive, multidisciplinary care and staged reconstructive surgeries. A key priority is therefore to minimize the surgical burden by consolidating procedures where possible and by optimizing each procedure to reduce need for revision surgeries (Fearon, 2003; Fearon and Podner, 2013). Fuller understanding of potential drivers for differential outcomes could provide valuable insights toward optimizing the care pathway.

Operative decisions in Apert hand syndactyly reconstruction are variable and multifactorial; numerous treatment algorithms have been proposed (Chang et al., 2002; Fadda et al., 2015; Fearon, 2003; Fearon and Podner, 2013; Guero, 2005; Guero et al., 2004; Pettitt et al., 2017; Raposo-Amaral et al., 2018; Van Heest et al., 1997). In light of inherent differences in syndactyly types among web spaces in Apert hands, the surgeon may choose different flap or incision approaches for individual web spaces on the same hand. Therefore, the aim of the present study was to characterize patterns of operative approaches for Apert hand syndactyly reconstruction across 15 years of cases at a major paediatric hospital, and to identify potential predictors of postoperative complications based on clinical presentations and reconstructive techniques. We hypothesized that patients with greater hand presentation severity, defined by the Upton classification, would experience higher incidence of postoperative complications. We also hypothesized that complication rates would correlate with operative approach, independent of hand type. Finally, we hypothesized that higher complication rates would result from the traditional approach to syndactyly reconstruction, using a dorsal rectangular commissural flap with zigzag flaps separating the fingers distally, in comparison to the simpler approach of interdigitating triangular commissural flaps and straight-line finger incisions.

## Methods

After Institutional Review Board approval (21-019312), this study reviewed a subset of a large database consisting of all upper- and lower-extremity syndactyly cases at a tertiary, 627-bed academic paediatric hospital from January 2007 to January 2022. The analysis captured all patients who underwent primary reconstruction of Apert hand syndactyly and were aged under 18 years at presentation to the clinic. Patients who did not receive primary reconstruction and follow-up care at our institution were excluded. Three independent reviewers extracted data from the medical record.

### Outcome measures

Clinical characteristics included Upton hand type and syndactyly type. Operative data included commissural flap design (e.g. interdigitating triangular flaps, rectangular flaps or Z-plasty), dorsal and volar incision technique along the distal fingers (e.g. straight-line incisions or zigzag flaps), skin graft donor site and attending surgeon. To account for phenotypic diversity and combinatory reconstructive techniques on a single hand, data were recorded at the level of individual web spaces.

The incidence of postoperative complications within 30 days of primary reconstruction were reviewed, along with late complications such as web creep, hypertrophic scarring, range-of-motion deficits, flexion contracture and angulation deformities. Revision surgeries were recorded, excluding any planned follow-up surgeries, such as clinodactyly repair. Time from primary reconstruction to last follow-up with the hand surgeon was determined, with data collection ending in April 2023 (more than 2 years after the latest primary surgery in the dataset). Demographic data were also collected.

### Data analysis

Analyses were conducted with Statistical Package for the Social Sciences (SPSS), version 28.0 (IBM Corporation, Armonk, NY, USA). Chi-square or Fisher’s exact tests were used to evaluate associations between hand/syndactyly classifications, reconstructive techniques and postoperative complications. Relative risk was determined for significant associations between hand type (grouped binarily post-hoc as Type III vs. other types) and postoperative complications. Relative risk was also determined for significant associations between reconstructive techniques and postoperative complications, excluding two Z-plasty commissuroplasties to achieve binary comparisons of rectangular versus interdigitating triangular commissural flaps and of zigzag finger flaps versus straight-line incisions. *p*-values less than 0.05 were considered statistically significant.

## Results

### Patient population

The analysis included 98 web space reconstructions in 17 patients. Only one patient had a relevant family history of syndactyly, and most of the cohort presented with comorbid diagnoses (Table 1). The cohort predominantly identified as White and non-Hispanic/Latino, with a 1.1:1 female-to-male ratio (Table 1). Patients typically presented to the clinic at approximately 1 month of age and underwent primary reconstruction at approximately 9 months (Table 1). The length of follow-up with the hand surgeon ranged from 6 months to 8 years after primary reconstruction (Table 1).

**Table 1.** Demographic and clinical characteristics (*n* = 17).

*Parameters*	
Upton hand type	
Type I (spade hand)	5 (29)
Type II (mitten hand)	8 (47)
Type III (rosebud hand)	4 (24)
Family history	1 (5.9)^a^
Sex	
Female	9 (53)
Male	8 (47)
Race	
White	12 (71)
Black	4 (24)
Other	1 (5.9)
Ethnicity	
Non-Hispanic/Latino	12 (71)
Hispanic/Latino	5 (29)
Comorbidities	9 (53)^b^
Age at presentation to the clinic (months)	1.3 (0.8–6.5)
Age at primary reconstruction (months)	9.1 (7.3–12.5)
Length of follow-up (months)	41.3 (SD 29.6)
Area Deprivation Index percentile	57.0 (SD 20.8)

Data are presented as *n* (%), mean (SD) or median (IQR).

a Syndactyly.

b Including choanal atresia, dysphagia, shoulder dysplasia, kyphosis, lordosis, infantile idiopathic scoliosis, cervical spinal fusion, eyelid lag, encephalocele, finger clubbing, patent foramen ovale.

IQR: interquartile range; SD: standard deviation.

All three Upton hand types were well-represented in this cohort (Table 1). One patient with Type III hands underwent bilateral amputation of the index finger. The 34 hands in the cohort therefore contained 134 total web spaces with 108 web space syndactylies and ultimately 98 web space reconstructions. Table 2 shows the distribution of syndactyly types.

**Table 2.** Characteristics of affected web spaces (*n* = 108).

	*n* (%)	Reconstruction performed	*p*
Syndactyly type			0.003
Simple incomplete	13 (12)	9 (69)	
Simple complete	24 (22)	22 (92)	
Complex	41 (38)	41 (100)	
Complicated	30 (28)	26 (87)	

Data are presented as *n* (%). *p*-values are from Fisher’s exact tests.

### Complication rates and relationship with presentation severity

**Figure 1.**
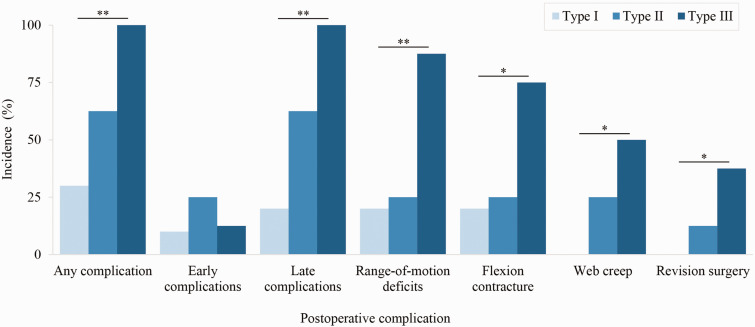
Incidence of postoperative complications increased significantly with increasing Upton hand type (severity). *p < 0.05; **p ≤ 0.01.

Postoperative complications occurred in 21 (62%) hands (Table 3) and 58 web spaces, affecting 71% of patients. Incidence of early complications (within 30 days) and late complications were 18% and 59% of hands, respectively (Table 3). Five hands in four patients required revision surgery due to web creep or skin necrosis in the first or second web spaces (Table 3). Upton hand type demonstrated a significant positive correlation with overall incidence of postoperative complications, and specifically was associated with late complications but not early complications (Figure 1, Table 4). Upton hand type was significantly correlated with range-of-motion deficits, flexion contracture, web creep and revision surgery (Figure 1, Table 4). Patients with Type III rosebud hands experienced double the risk of postoperative complications compared to patients with Type I or II hands, with more than double the risk of late complications (Table 4). Patients with Type III hands experienced nearly quadruple the risk of postoperative range-of-motion deficits and more than triple the risk of postoperative flexion contracture compared to Types I/II (Table 4). Risk of web creep and revision surgery increased significantly with increasing Upton hand type severity, and post-hoc analyses indicated that the risk difference applied ordinally to Types II and III hands rather than Type III specifically (Table 4; significance was lost in binary comparisons).

**Table 3.** Incidence of postoperative complications per hand (*n* = 34).

	*n* (%)
Any complication	21 (62)
Early complications	6 (18)
Infection	3 (8.8)
Skin necrosis (eschar)	1 (2.9)
Granulation/graft failure	1 (2.9)
Early dressing change	1 (2.9)
Late complications	20 (59)
Range-of-motion deficits	13 (38)
Flexion contracture	12 (35)
Web creep	8 (24)
Angulation deformity	5 (15)
Hypertrophic scarring/scar contracture	4 (12)
Delayed wound healing	2 (5.9)
Revision surgery	5 (15)
Web creep revision	4 (12)
Eschar debridement	1 (2.9)

**Table 4.** Upton hand type as a predictor of postoperative complications.

Upton hand type	Type I (*n* = 10)	Type II (*n* = 16)	Type III (*n* = 8)	*p*	Relative risk (95% CI) for Type III hands
Any complication	3 (30)	10 (63)	8 (100)	0.01	2.0 (1.4 to 2.9)
Early complications	1 (10)	4 (25)	1 (13)	0.62	–
Late complications	2 (20)	10 (63)	8 (100)	0.002	2.2 (1.4 to 3.3)
Range-of-motion deficits	2 (20)	4 (25)	7 (88)	0.005	3.8 (1.8 to 8.0)
Flexion contracture	2 (20)	4 (25)	6 (75)	0.04	3.3 (1.5 to 7.3)
Web creep	0 (0.0)	4 (25)	4 (50)	0.04	3.3 (1.0 to 10.1)^a^
Revision surgery	0 (0.0)	2 (13)	3 (38)	0.04	4.9 (1.0 to 24.3)^a^

Data are presented as *n* (%). *p*-values are from Fisher’s exact tests. Relative risk was determined for significant associations, with hand type grouped binarily post-hoc as Type III versus other types.

a Web creep and revision surgery lost significance (both Fisher’s exact *p* = 0.07) after grouping Types I/II.

CI: confidence interval.

### Operative approach and relative risk of complications

**Figure 2.**
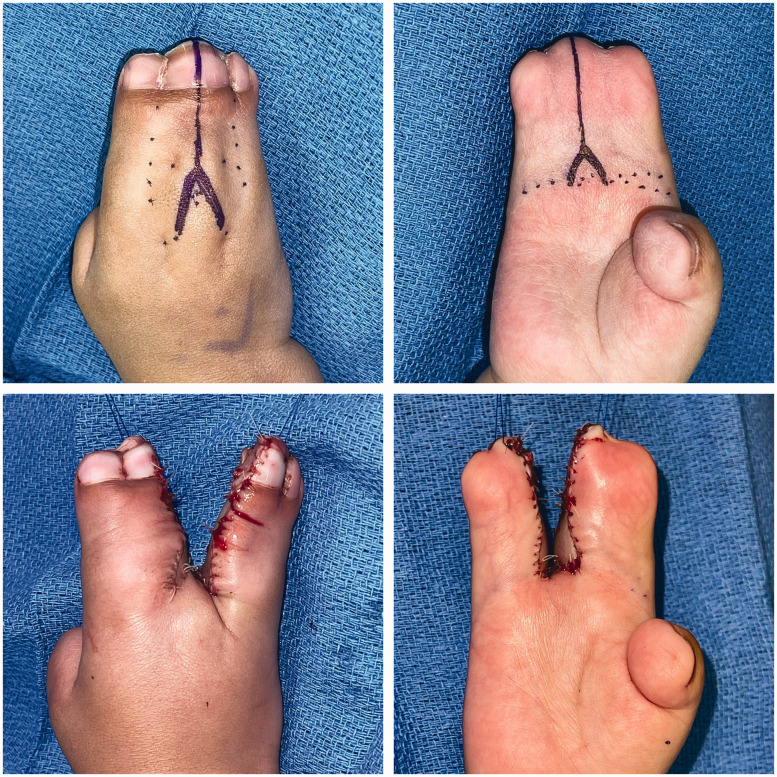
Stage 1 reconstruction of a Type I Apert hand, using interdigitating triangular commissural flaps with dorsal and volar straight-line incisions along the fingers.

**Figure 3.**
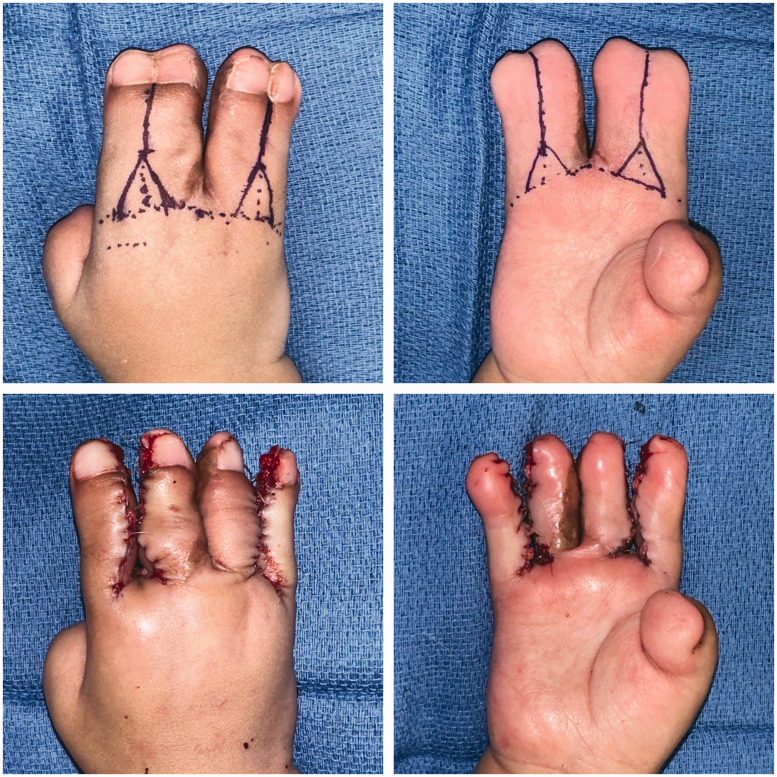
Stage 2 reconstruction of a Type I Apert hand, using interdigitating triangular commissural flaps with dorsal and volar straight-line incisions along the fingers.

Syndactyly reconstructions in this cohort were performed by three attending hand surgeons, with Level 3–4 expertise according to Tang and Giddin’s (2016) criteria. Operative approaches including choice of commissural flap, dorsal and volar finger flap/incision, and skin graft donor site generally did not follow clear trends by syndactyly type or web space number (Tables 5 and 6). In total, 98 web spaces (91% of those affected) were reconstructed. All complex syndactylies underwent reconstruction, whereas simple incomplete syndactylies were least likely to undergo reconstruction (Table 2). At our institution, fused bones in complicated or complex syndactylies are separated using an osteotome or knife; bone involvement generally does not affect the soft-tissue treatment or choice of flap. The procedural goals, especially the goal of achieving a five-digit hand, are the same regardless of bone involvement. Syndactyly type did not correlate significantly with complications in this cohort. The most common approach was interdigitating triangular flaps at the web commissure with straight-line incisions along the fingers dorsally and volarly (Figures 2 and 3). The groin was the most common donor site for full-thickness skin grafts, though skin grafts from the lower abdomen were relatively more common in syndactylies with bony fusion (43% of complex or complicated syndactylies versus 16% of simple syndactylies; *p* = 0.009).

**Table 5.** Operative approach by web syndactyly type (*n* = 98).

Syndactyly type	Simple incomplete	Simple complete	Complex	Complicated	Overall
Commissural flap design					
Interdigitating triangular	5 (56)	22 (100)	33 (80)	24 (92)	84 (86)
Rectangular	2 (22)	0 (0.0)	8 (20)	2 (7.7)	12 (12)
Z-plasty	2 (22)	0 (0.0)	0 (0.0)	0 (0.0)	2 (2.0)
Dorsal finger incision design					
Straight-line	7 (78)	22 (100)	37 (90)	26 (100)	92 (94)
Zigzag	0 (0.0)	0 (0.0)	4 (9.8)	0 (0.0)	4 (4.1)
None (Z-plasty)	2 (18)	0 (0.0)	0 (0.0)	0 (0.0)	2 (2.0)
Volar finger incision design					
Straight-line	7 (78)	19 (86)	36 (88)	24 (92)	86 (88)
Zigzag	0 (0.0)	3 (14)	5 (12)	2 (7.7)	10 (10)
None (Z-plasty)	2 (18)	0 (0.0)	0 (0.0)	0 (0.0)	2 (2.0)
Skin graft donor site					
Groin	7 (78)	19 (86)	27 (66)	11 (42)	64 (65)
Lower abdomen	2 (18)	3 (14)	14 (34)	15 (58)	34 (35)

Data are presented as *n* (%).

**Table 6.** Operative approach by web space (*n* = 98).

Web space	1	2	3	4	Overall
Commissural flap design					
Interdigitating triangular	7 (64)	23 (79)	29 (94)	25 (93)	84 (86)
Rectangular	2 (18)	6 (21)	2 (6.5)	2 (7.4)	12 (12)
Z-plasty	2 (18)	0 (0.0)	0 (0.0)	0 (0.0)	2 (2.0)
Dorsal finger incision design					
Straight-line	9 (82)	27 (93)	31 (100)	25 (93)	92 (94)
Zigzag	0 (0.0)	2 (6.9)	0 (0.0)	2 (7.4)	4 (4.1)
None (Z-plasty)	2 (18)	0 (0.0)	0 (0.0)	0 (0.0)	2 (2.0)
Volar finger incision design					
Straight-line	9 (82)	26 (90)	29 (94)	22 (82)	86 (88)
Zigzag	0 (0.0)	3 (10)	2 (6.5)	5 (19)	10 (10)
None (Z-plasty)	2 (18)	0 (0.0)	0 (0.0)	0 (0.0)	2 (2.0)
Skin graft donor site					
Groin	6 (55)	18 (62)	26 (84)	14 (52)	64 (65)
Lower abdomen	5 (46)	11 (38)	5 (16)	13 (48)	34 (35)

Data are presented as *n* (%).

**Figure 4.**
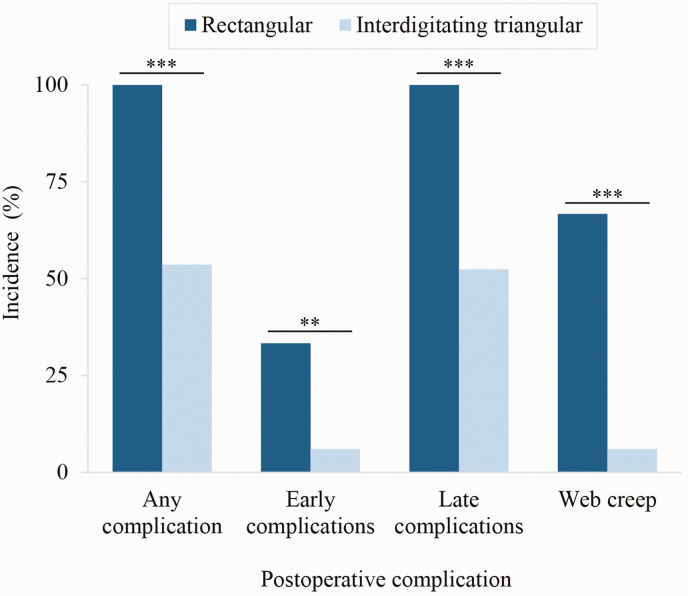
Web commissuroplasties with a rectangular flap showed significantly higher incidence of postoperative complications than interdigitating triangular flaps. **p ≤ 0.01; ***p ≤ 0.001.

**Figure 5.**
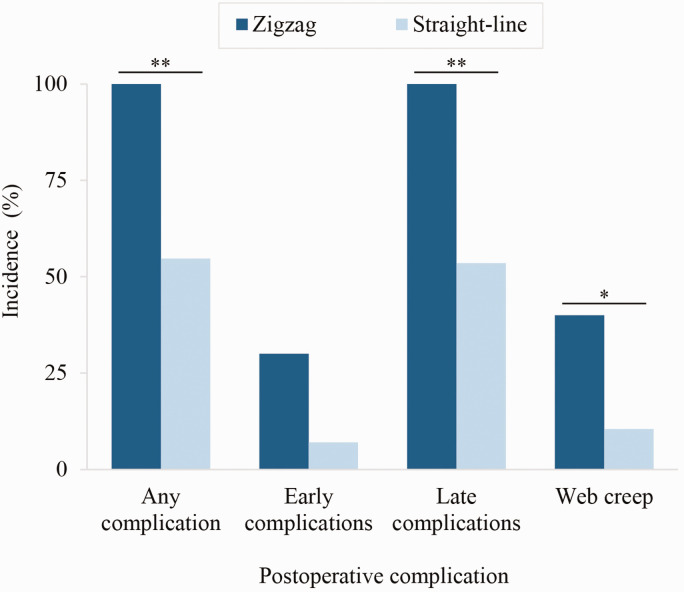
Zigzag volar finger flaps showed significantly higher incidence of postoperative complications than straight-line incisions. *p < 0.05; **p ≤ 0.01.

Web spaces reconstructed with a rectangular commissural flap showed significantly higher incidence of complications than interdigitating triangular flaps (Table 7). Compared to interdigitating triangular flaps, rectangular commissural flaps showed nearly double the risk of yielding any postoperative complications and more than 11 times greater risk of web creep (Figure 4, Table 7). Fingers separated with zigzag volar finger flaps showed nearly double the risk of postoperative complications compared to straight-line incisions (Figure 5, Table 7). Specifically, zigzag volar finger flaps demonstrated almost quadruple the risk of web creep (Figure 5, Table 7). Rectangular commissural flaps showed significantly elevated risk of both early complications and late complications (Figure 4, Table 7); zigzag volar finger flaps showed significantly elevated risk of late complications (Figure 5, Table 7). Confounding between hand type and operative approach cannot fully explain these findings, as Upton hand type did not correlate significantly with commissural flap design, nor with volar or dorsal finger incision technique. Syndactyly type, web space number, dorsal finger incision technique and skin graft donor site were not significantly associated with incidence of complications in this cohort. Incidence of hypertrophic scarring/scar contracture did not differ significantly by finger incision technique.

**Table 7.** Operative approaches as risk factors for postoperative complications.

Web-commissure flap design	Rectangular (*n* = 12)	Interdigitating triangular (*n* = 84)	*p*	Relative risk (95% CI)^a^
Any complication	12 (100)	45 (54)	0.001	1.9 (1.5 to 2.3)
Early complications	4 (33)	5 (6.0)	0.01	5.6 (1.7 to 18.0)
Late complications	12 (100)	44 (52)	0.001	1.9 (1.6 to 2.3)
Web creep	8 (67)	5 (6.0)	<0.001	11.2 (4.4 to 28.7)
Volar finger incision design	Zigzag (*n* = 10)	Straight line (*n* = 86)	*p*	Relative risk (95% CI)^a^
Any complication	10 (100)	47 (55)	0.005	1.8 (1.5 to 2.2)
Early complications	3 (30)	6 (7.0)	0.050	–
Late complications	10 (100)	46 (54)	0.005	1.9 (1.5 to 2.3)
Web creep	4 (40)	9 (11)	0.03	3.8 (1.4 to 10.2)

Data are presented as *n* (%). *p*-values are from Fisher’s exact tests. Operative approaches did not significantly predict incidence of specific complications other than web creep.

a Relative risk represents rectangular versus interdigitating triangular web-commissure flaps and zigzag finger flaps versus straight-line incisions; 2 Z-plasty commissuroplasties were excluded from this analysis to achieve binary comparisons.

CI: confidence interval.

Operative techniques by attending surgeon were compared to assess possible confounding. This analysis was descriptive due to low statistical power. Rectangular commissural flaps were used in six hands (three patients) by two different surgeons; all cases resulted in at least one late complication per web space with or without early complications. Zigzag volar finger flaps were used in seven hands (four patients) by two different surgeons; all cases resulted in at least one late complication per web space with or without early complications. Therefore, differences in complication rates by reconstructive technique did not appear to result from provider differences.

### Postoperative management

Nearly all postoperative dressings (all surgeries for 16/17 patients) involved antibiotic ointment on the grafts and sutures, non-adhesive Adaptic™ gauze in the web spaces, a double layer of sterile Webril® undercast padding between each digit and Webril® wrapping around the hands. The hand was either immobilized in a long-arm fiberglass mitten cast, typically with the elbow at 90° and the thumb exposed, or wrapped in soft self-adherent Coban™ from the fingertips to the elbow. For one patient, a cotton bolster soaked in mineral oil was placed in the web space with additional sterile dressings in lieu of the typical dressing combination. This was the only case that required an early dressing change due to bleeding at the dressing site.

## Discussion

Fearon (2003) advocated for straight-line incisions rather than zigzag flaps along the fingers and triangular interdigitating flaps rather than a long dorsal rectangular flap at the commissure, which was practice-changing for many providers. In this study, we were able to confirm that Fearon’s approach to Apert hand syndactyly reconstruction (Fearon, 2003) is associated with lower complication rates, contributing to the evidence base for ongoing optimization of the care pathway. Fearon’s recommendations produced a paradigm shift (Fearon, 2003; Pettitt et al., 2017) away from previous Apert hand treatment algorithms that aimed for a three- or four-digit hand (Chang et al., 2002; Van Heest et al., 1997). Yet controversy persists over various factors, including incision technique, choice of skin graft donor site, the appropriate number and timing of operative sessions, prioritization of border digits and acceptability of finger amputation (Fearon, 2003; Fearon and Pettitt et al., 2017; Podner, 2013; Raposo-Amaral et al., 2018). A multicentre survey published 14 years after Fearon’s recommendations revealed that many surgeons still favour alternative flap designs (Pettitt et al., 2017).

To our knowledge, no studies have directly compared postoperative outcomes between Fearon’s protocol and other approaches. We analysed surgical patterns and outcomes at the level of the web space to lend a novel, detailed perspective on the appropriateness of different operative techniques.

In this 15-year series, surgeons favoured interdigitating triangular flaps at the web commissure over a long dorsal rectangular flap, consistent with Fearon’s recommendations (Fearon, 2003). The rectangular commissural flap was associated with significantly higher incidence of web creep and of complications overall. In web spaces reconstructed with rectangular versus interdigitating triangular commissural flaps, patients experienced nearly double the risk of developing a postoperative complication and more than 11 times greater risk of developing web creep. After stratifying between early and late complications, differences remained significant for both subsets of complications. Further analysis also showed that commissural flap design was not significantly associated with Upton hand type, indicating that these results were not simply due to confounding with underlying severity. These results suggest that interdigitating triangular flaps at the web commissure are preferable in most Apert hand cases. We speculate that the dual contributions from dorsal and volar interdigitating triangular flaps may yield less tension and scarring at the commissure than a single dorsal rectangular flap, in turn reducing web creep and other complications.

**Figure 6.**
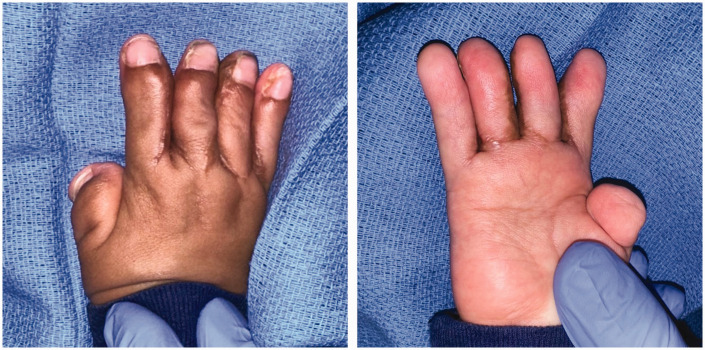
Outcome at 6-month follow-up from stage 1 and 3-month follow-up from stage 2 reconstruction of the hand shown in Figures 2 and 3, demonstrating minimal scar visibility.

In our cohort, surgeons also favoured dorsal and volar straight-line incisions along the distal fingers over the zigzag technique. In line with Fearon’s recommendations, straight-line incisions are aesthetically preferable because they close along the mid-lateral line, which minimizes scar visibility and protects the neurovascular bundle (Fearon, 2003; Raposo-Amaral et al., 2018). Zigzag flaps are visible on the dorsal and volar surfaces of the fingers; though they may reduce the risk of scar contracture in fingers with mobile interphalangeal joints, they are of no benefit in Apert syndactyly reconstructions between fingers with symphalagism (Cronin, 1956; Fearon, 2003). Although we note that all cases (eight web spaces on four total hands) of hypertrophic scarring/scar contracture developed after straight-line incision in this cohort, incidence did not differ significantly by finger incision technique. However, zigzag volar finger flaps were associated with significantly higher incidence of web creep and of complications overall. In web spaces divided with zigzag volar finger flaps versus straight-line incisions, patients experienced nearly double the risk of developing a postoperative complication and, more specifically, nearly quadruple the risk of developing web creep. Upton hand type did not correlate significantly with volar finger incision technique, indicating that zigzag flaps independently contribute to complications. In light of these findings, we endorse the simpler surgical approach of volar and dorsal interdigitating triangular flaps for the commissure, and straight-line incisions for the volar and dorsal fingers in most Apert hand cases (Figures 2, 3 and 6). We also recommend a combination of antibiotic ointment, Adaptic™ gauze and Webril® wrapping with either a hard or soft cast for postoperative dressings, which did not lead to early dressing change complications.

Our analysis also confirmed that Upton hand type is an important consideration for the incidence of postoperative complications. Providers should be aware that, based on this study, patients with Type III rosebud hands experience more than triple the risk of postoperative range-of-motion deficits and flexion contracture than patients with Type I or II hands, and double the risk of postoperative complications. The incidence of web creep and revision surgery rises significantly with increasing Upton hand type severity; we note that no patients with Type I hands demonstrated web creep or need for revision. Five hands (12%) from four patients (24%) in this cohort required revision surgery; this outcome was consistent with previously reported revision rates, which have been in the range of 13%–32% (Chang et al., 2002; Guero, 2005; Raposo-Amaral et al., 2018). Apert syndrome causes global hand pathology; as such, Apert hand syndactylies often warrant different management than non-syndromic hands with multiple syndactylies. While we recommend interdigitating triangular commissural flaps and straight-line finger incisions for operable web spaces, we note that syndactyly reconstruction may not be appropriate for all affected web spaces in these complex hands. Ongoing clinical practice guideline development should devote special focus to patients with greater degrees of Apert hand severity, particularly Type III hands, regarding preventive measures for adverse postoperative outcomes. For example, Theman et al. (2018) and Raposo-Amaral et al. (2018) suggested that a modified three-stage reconstruction strategy may be more appropriate for Type III hands, while Fearon’s two-stage approach may be adequate for Types I and II. Theman et al. (2018) used limited osteotomies during stage 1 to reposition the overlapping, concave central digits into a single plane, providing better soft tissue distribution and facilitating subsequent flap designs for a five-digit hand.

The present study has some limitations, including its retrospective nature. Although our institution is a busy tertiary paediatric centre, the rarity of Apert syndrome and the fact that this study only included patients who had their primary hand treatment at our centre restricted the sample size to 17 patients, thus limiting the statistical power of our analyses. The sample was underpowered to detect associations between demographic variables and clinical outcomes. Future research on how social determinants of health may influence prognoses for patients with Apert syndrome is warranted, potentially pooling data from multiple institutions. The medical record did not capture the location of digital artery bifurcation, precluding analysis of its possible relationship with complication rates. Lastly, our database lacked prospective measures of longitudinal functional outcomes. While postoperative complications are a crucial metric of treatment success, long-term data on hand use and quality of life would be a valuable complement in future studies (Raposo-Amaral et al., 2019).

This analysis identified specific factors associated with complications after Apert hand syndactyly reconstruction, spotlighting important directions for further study as providers seek to optimize the Apert hand care pathway. Patients with greater degrees of Apert hand severity, particularly Type III hands, may benefit from additional proactive measures to minimize postoperative complications. Our results also warrant prospective comparative research on flap design at the web commissure and incision technique along the volar distal fingers, which may help establish consensus on best clinical practice.
